# Patients with Obesity and a History of Metformin Treatment Have Lower Influenza Mortality: A Retrospective Cohort Study

**DOI:** 10.3390/pathogens11020270

**Published:** 2022-02-19

**Authors:** Tammy H. Cummings, Joseph Magagnoli, James W. Hardin, S. Scott Sutton

**Affiliations:** 1 Dorn Research Institute, Columbia Veterans Affairs Health Care System, Columbia, SC 29209, USA; harris68@mailbox.sc.edu (T.H.C.); magagnol@mailbox.sc.edu (J.M.); jhardin@sc.edu (J.W.H.); 2 Department of Clinical Pharmacy and Outcomes Sciences, College of Pharmacy, University of South Carolina, Columbia, SC 29208, USA; 3 Department of Epidemiology & Biostatistics, University of South Carolina, Columbia, SC 29208, USA

**Keywords:** influenza, obesity, metformin, T-cell restoration, drug repurposing

## Abstract

Background: Obesity is a risk factor for the development of influenza by leading to a chronic inflammatory state and T-cell dysfunction. Based upon preclinical research, metformin has influenza activity by restoring T-cell function and improving the immune response. Objective: We aimed to evaluate the potential drug repurposing of metformin for the management of influenza among patients with obesity utilizing national claims data in an electronic health record database. Methods: The VA Informatics and Computing Infrastructure (VINCI) was utilized to obtain individual-level information on demographics, administrative claims, and pharmacy dispensation. A cohort was created among individuals with laboratory confirmed diagnosis of influenza with a diagnosis of fever, cough, influenza, or acute upper respiratory infection in an outpatient setting. The study outcome was death after diagnosis of influenza. Cohorts were formed using diabetes status and metformin exposure prior to a positive influenza diagnosis. Hazard ratios for mortality were estimated using a cox proportional hazards model adjusting for baseline covariates and a sub-analysis was conducted utilizing propensity score matching. A greedy nearest neighbor algorithm was utilized to match 1 to 1 non-metformin diabetic controls and non-diabetic controls to diabetic patients receiving metformin. Results: A total of 3551 patients met the inclusion criteria and were evaluated in our study. The cohorts consisted of 1461 patients in the non-diabetic cohort, 1597 patients in the diabetic / metformin cohort, and 493 patients in the diabetic no metformin cohort. Compared to non-diabetic patients, diabetic patients with metformin had a lower rate of death (aHR 0.78, 95% CI 0.609–0.999). There was not a statistical difference between the non-diabetic patients and the diabetic patients without metformin (aHR 1.046, 95% CI 0.781–1.400). The propensity score matched cohorts revealed consistent results with the primary analysis. Conclusion: Our results demonstrated patients with obesity and a history of metformin treatment have lower influenza mortality.

## 1. Introduction

Influenza is a contagious viral respiratory tract infection that causes significant morbidity and mortality. The worldwide influenza burden causes up to 500,000 deaths and 5 million infections annually [1]. Influenza also poses a potential public health threat because of the ability to cause pandemics (e.g., 1918 influenza, 2009 H1N1) [2]. Traditional risk factors for influenza complications are well documented and include immunosuppression, children younger than 5 years of age and adults 65 years of age and older; however, a new risk factor was added after the 2009 influenza pandemic to include obesity [3,4]. The identification of obesity as an independent risk factor for influenza morbidity and mortality is of significant concern because there are nearly 500 million individuals with obesity worldwide. Influenza vaccination remains a key component to prevent influenza infections; however, vaccinated individuals with obesity have a higher risk of influenza and influenza-like illness compared to vaccinated healthy adults, despite equal serologic response to the vaccine [5,6]. Mechanistically, there is an alteration of T cells in patients with obesity that impairs the response to influenza infections [7,8,9]. Importantly, altered T cell function among mice did not improve with weight loss [10]. As influenza vaccination or weight loss among obesity does not provide similar influenza outcomes compared to non-obesity, repurposing of existing medications to address this clinical disparity is an urgent priority. A preclinical study demonstrated that metformin can improve or restore T cell function and could serve as a potential treatment for influenza among patients with obesity [9]. Therefore, we sought to demonstrate a unique integration of large-scale patient level longitudinal data testing the hypothesis from published preclinical research on metformin as an antiviral for influenza. Specifically, we examined metformin usage and mortality rates among patients with obesity and laboratory confirmed influenza among a national cohort of patients.

## 2. Methods

### 2.1. Data

This drug disease cohort study was conducted using data from the U.S. Department of Veterans Affairs. The VA Informatics and Computing Infrastructure (VINCI) was utilized to obtain individual-level information on demographics, administrative claims and pharmacy dispensation. The study was conducted in compliance with the Department of Veterans Affairs requirements and received Institutional Review Board and Research and Development Approval. The study utilized inpatient and outpatient claims coded with International Classification of Diseases (ICD) revision 9-CM, revision 10-CM, Current Procedure Terminology (CPT) and pharmacy data.

### 2.2. Cohort Selection

A cohort was created among individuals with laboratory confirmed diagnosis of influenza and ICD-9/10 diagnosis codes of fever, cough, influenza or acute upper respiratory infection in an outpatient setting. Influenza status was classified by an initial RNA laboratory result that was extracted from the VA laboratory data utilizing Logical Observation Identifiers Names and Codes (LOINC) (Table S6). The date of a positive influenza lab result was the study index and index dates range from January 2011 to December 2019. Patients were followed from index to death or end of study period (1 December 2020). Patients were included in the study if they (1) were at least 18 years old at diagnosis; (2) had greater than one year between VA enrollment and index; (3) had a flu vaccine 9 months prior to index; (4) had a body mass index (BMI) of greater than or equal to 30; (5) were diagnosed in a primary care or emergency department setting; and (6) were not transferred to the emergency department.

### 2.3. Study Outcome

The study outcome was death occurring after diagnosis of influenza yet before end of study. Date of death was extracted from the VA vital status files. The time from index until death was utilized as the outcome variable.

### 2.4. Medication Exposure

Cohorts were formed using diabetes status and metformin exposure prior to a positive influenza diagnosis. Diabetes was coded using ICD-9 or ICD-10 codes of 249.x, 250.x, E10.x, E11.x. Diabetics with at least one outpatient pharmacy dispense of metformin prior to positive influenza diagnosis were classified into metformin exposed cohort. Diabetics without metformin exposure were classified into the diabetic non metformin exposed cohort. Patients with no diagnoses of diabetes or metformin exposure were classified into the non-diabetic cohort.

### 2.5. Baseline Data

We extracted data on baseline demographic and comorbidity. Demographic and clinical characteristics included age, sex, race/ethnicity and year of visit. Comorbid conditions include all conditions included in the Charlson comorbidity index, hypercholesterolemia, hyperlipidemia, hypertriglyceridemia, hypertension, smoking, ischemic heart disease and heart disease.

### 2.6. Statistical Analysis

The statistical analysis occurred in three steps. First, we generate summaries of the baseline demographic and clinical characteristics for each cohort and evaluate differences between cohorts using chi-square test or analysis of variance (ANOVA). Second, we estimate the hazard ratio for death after influenza diagnosis using a cox proportional hazards model while adjusting for baseline covariates. Last, to evaluate the robustness of our findings, we utilized propensity score matching to minimize observable differences between cohorts. We fit a logistic regression model including demographic and comorbid factors listed in Table 1 to predict treatment with metformin. We then utilized a greedy nearest neighbor algorithm to match 1 to 1 non-metformin diabetic controls as well as 1 to 1 non-diabetic controls. We use the largest standardized difference among the three cohorts as a measure of closeness. Data management and analysis was performed using SAS (SAS Institute Inc., SAS Enterprise Guide 8.2, Cary, NC, USA).

## 3. Results

A total of 3551 patients met the inclusion criteria and were evaluated in our study. The cohorts consisted of 1461 patients in the non-diabetic cohort, 1597 patients in the diabetic/metformin cohort and 493 patients in the diabetic no metformin cohort. The cohorts had a mean age ranging from 60 to 69 years of age and were predominately males. Additional baseline demographics and clinical characteristics are included in Table 1 and consist of the Charlson Comorbidity Index, hemoglobin A1c and index year. Among the diabetic patients, the metformin cohort had the lowest mortality rate (12.4%) compared to the non-metformin diabetic cohort (18.66%). The non-diabetic cohort had the lowest overall mortality rate at 8.08% (Table 2). There are many variables that can influence mortality rates, including mortality rates with patients diagnosed with influenza. Therefore, we estimated the hazard ratio for death after influenza diagnosis using a cox proportional hazards model while adjusting for baseline covariates (Table 3). The results demonstrated that compared to a reference group of non-diabetic patients, diabetic patients with metformin had a lower rate of death (aHR 0.78, 95% CI 0.609–0.999). There was not a statistical difference between the non-diabetic patients and the diabetic patients without metformin (aHR 1.046, 95% CI 0.781–1.400). There were several covariates that were statistically significant in the multivariable statistical model and include age, Charlson comorbidity index, hyperlipidemia, smoking, heart disease and index year. Importantly, this finding is among cohorts of patients with a BMI greater than or equal to 30 and receipt of a flu vaccine within 9 months of influenza diagnosis.

## 4. Discussion

Influenza is a contagious viral respiratory infection that affects millions of individuals annually. Risk factors for epidemic/endemic influenza complications are well known and include the elderly, children and immunocompromised patients [7,11,12,13]. However, following the first pandemic influenza outbreak of the 21st century (2009 H1N1), a BMI greater than or equal to 30.0 kg/m^2^ was identified as an independent risk factor for increased morbidity and mortality. Obesity has been a well-known risk factor for chronic diseases and is now recognized as a risk factor for acute infectious diseases [7,14,15]. Because there are over 500 million patients with obesity worldwide and patients with obesity outnumber underweight adults, influenza among patients with obesity is a public health area that needs urgent attention. Specifically, new pharmacologic treatment options among patients with obesity are needed because vaccinated adults with obesity have twice the risk of influenza or influenza-like illness compared to vaccinated healthy-weight adults [5]. Over-nutrition that results in obesity causes a chronic state of inflammation and impairs the immune response to influenza infection and influenza vaccination through alteration of the T cells [6,7,16]. Therefore, we set out to evaluate the utilization of metformin on mortality rates among patients with obesity diagnosed with influenza. Utilizing an administrative claim database, we demonstrated that diabetic patients with obesity and prescribed metformin had improved overall survival after an influenza diagnosis confirmed via polymerase chain reaction (PCR). This is the first, to our knowledge, evaluation among patients utilizing metformin documenting an influenza treatment response. Our research selected and evaluated metformin based upon preclinical data [7,9,17]. Specifically, published data suggest that an alteration in the metabolic profile of T cells in individuals with obesity impairs the activation of the adaptive immune cells [7,9]. T cell function and metabolism are linked, and metabolic reprogramming of T cells can alter T cell differentiation, survival and function [18,19]. Obesity impairs the protective immunity of T cells and by targeting T cell metabolism with the anti-diabetic medication metformin can improve the immune response [9]. The collective preclinical information on obesity, influenza, T cells and metformin demonstrates the potential of improving (or restoring) T cell function to ultimately improve influenza infection outcomes. The addition of our real-world data among obese patients diagnosed with PCR confirmed influenza and improved overall survival among patients receiving metformin supports continued evaluation of metformin as an antiviral or targeting T cell restoration as a treatment for influenza.

There are important limitations to our drug disease observational study intrinsic to all health insurance claims database analyses, particularly proper documentation and coding [20,21]. The study utilized data from the Veterans Affairs Informatics and Computing Infrastructure (VINCI) and did not utilize registries, other data sets or potential data from the Veterans Choice Program. An additional study limitation is the population was predominately white males; therefore, our findings may not be generalizable to patients of different age groups or races. Specifically, the cohort of patients may have different proportions of comorbidities, and although we controlled for many comorbidities within our statistical models, the results may not be reflective of non-Veterans Affairs populations. As with all retrospective studies, including ours, treatment was not randomized and differences among the treatment groups could influence the outcome. Therefore, we conducted a sub-analysis utilizing propensity score matching to assemble cohorts of patients with similar baseline characteristics with the attempt to reduce possible bias in estimating the treatment effect. We exact matched on non-ischemic heart disease, while including all other covariates in the propensity score. Table S1 lists the baseline demographic and clinical characteristics of the matched samples. Each cohort had 456 patients and the results of the propensity score matched statistical model is consistent with our primary analysis. Among diabetic patients, the metformin cohort had a lower mortality rate (7.9%) compared to the non-metformin diabetic cohort (16.23%). However, unlike the initial model, the non-diabetic cohort a higher mortality rate (12.5%) than the diabetic metformin cohort but lower than the diabetic no metformin cohort (Table S2). We then estimated the hazard ratio for death after influenza diagnosis using a cox proportional hazards model while adjusting for baseline covariates. The results are consistent with the primary statistical model and demonstrated diabetic patients with metformin had a lower rate of death (aHR 0.590, 95% CI 0.384–0.905) compared to non-diabetic patients (Table S3). Like the initial statistical model, there was not a statistical difference between the non-diabetic patients and the diabetic patients without metformin (aHR 1.154, 95% CI 0.805–1.654). We also conducted another sub-analysis by excluding any patients who had a positive PCR test for COVID-19 during the study period. Table S7 lists the baseline demographic and clinical characteristics of the sample of patients without a positive COVID-19 test. Among diabetic patients, the metformin cohort had a lower mortality rate (12.73%) compared to the non-metformin diabetic cohort (19.41%). However, similar to the initial model, the non-diabetic cohort a lower mortality rate (8.29%) than the diabetic cohorts (Table S8). Like our original and propensity score matched results, diabetic patients with metformin had a lower rate of death (aHR 0.778, 95% CI 0.608–0.996) compared to non-diabetic patients (Table S9). Overall, the results of propensity score matching, and other sub-analysis (excluding COVID-19 patients) utilized within our study, increased the internal validity of the findings within our main analyses. However, all statistical modeling approaches are subject to inherent assumptions and limitations, including propensity score-based methods. Ultimately, prospective randomized trials provide the best insights into causality. Although our study exhibits limitations that are common to retrospective analysis, our study findings complimented with published preclinical data with metformin demonstrate the need to continue evaluation of T cell restoration for the treatment of influenza among patients with obesity. Despite our limitations, our study included many strengths, such as the utilization of a large-scale, patient-level data collected as part of routine patient care. The unique strength of routine healthcare data makes them ideal for testing or validating data generated from preclinical studies. The availability of clinical factors within real world data allows for covariate adjustment to minimize confounding [22,23,24,25]. Specifically to our study, we utilized electronic health records among a large sample size consisting of a nationwide population that include the availability of actual pharmacy dispensation data. We studied patients in an integrated national healthcare system; therefore, the data are less susceptible to biases of single-center or regional studies.

The outcome of this study was the mortality rate after an influenza diagnosis. Definitions within retrospective studies are critical to the results as changing of a definition can impact the results. The appropriate timeline to evaluate mortality after influenza can be difficult to define. Although it is possible to die from acute influenza, many cases of influenza death are from complications, including late complications, and not the virus itself. There has been an association between laboratory confirmed influenza and complications that involve organ systems outside the respiratory tract [26]. For example, cardiovascular disease and influenza have been associated and epidemiologic studies demonstrating an increase in cardiovascular deaths after influenza infection [27,28,29]. However, we cannot rule out the role of metformin impacting other organ systems or disease processes unrelated to influenza ultimately leading to our results of decreased mortality. For example, among pre-diabetic patients with a BMI > 35, metformin had a lower incidence of coronary heart disease [30]. Additionally, there could be an association between obesity and type 2 diabetes that are involved in the influenza disease process and not just obesity alone. However, metformin has also demonstrated, in a pre-clinical study, the ability to inhibit metabolic changes and dysfunction of select T cells that may be involved in tumor progression and susceptibility to virus infection in type 2 diabetes [31].

Because influenza is associated with acute and chronic complications that can impact mortality, we evaluated all-cause mortality at any time. As mentioned, the timeframe of defining mortality is important in the interpretation of our results. Our findings demonstrate a reduction in mortality among patients with obesity and confirmed influenza that have received an influenza vaccine within the previous 9 months and receiving metformin. However, we feel that it is critical in interpretation of this repurposing study to be transparent about the definition of the mortality timeline. Our definition was designed to capture acute and long-term mortality. However, it is possible that a non-influenza complication was captured among the overall mortality results as metformin also improves non-influenza related conditions [32,33,34]. Therefore, we conducted a sub-analysis to evaluate mortality rates at 30- and 60-days post influenza diagnosis. Consistent with the original analysis, patients with no diabetes had the lowest 30- and 60-day mortality rate. This finding is expected as the Charlson comorbidity index is significantly lower among the non-diabetic cohort. Among the diabetic cohorts, patients prescribed metformin had a lower 30- and 60-day mortality rate (Table S4). The cox proportional hazards model did not find a statistical association at 30- or 60-day mortality (Table S5). However, the overall number of deaths among all three cohorts at 30- and 60-days were very low. Finally, because we evaluated an administrative claims data base for our study, we are not able to evaluate the impact of changes within the influenza virus over time. A change in the influenza virus over time could significantly impact our results. We attempted to control for this by requiring influenza vaccination within 9 months prior to the influenza diagnosis. Controlling for influenza vaccine status supports the overall finding because the vaccine is developed based upon the expected influenza strains circulating and because vaccination remains the primary means of influenza prevention.

Importantly, we are not advocating for the clinical utilization of metformin, at this time, for the treatment of influenza. The intent of this study was to demonstrate the potential of metformin among patients with obesity for the treatment of influenza. As our team utilized an administrative claims database to evaluate metformin for influenza treatment, we are dependent upon patients receiving metformin for another cause and in this study the cause of metformin receipt was management of diabetes, not influenza. However, the results of our study demonstrate a signal for utilizing metformin in the treatment of influenza. Further research is warranted to fully understand this relationship before utilization can be recommended in clinical practice.

## 5. Conclusions

Obesity is an independent risk factor for increased morbidity and mortality in response to influenza infection. Therefore, we aimed to evaluate metformin utilization for the management of influenza utilizing a large, national claims and electronic health record database. Our results demonstrated a decreased mortality rate for patients with obesity diagnosed with influenza and treated with metformin. Further research on metformin is warranted for the treatment of influenza.

## Figures and Tables

**Table 1:** Baseline demographic and comorbid characteristics.

		Diabetes				
Variable		No Metformin N = 493	Metformin Exposed N = 1597	Non-Diabetes N = 1461	*p*-Value *	Standardized Difference
Sex	Female	26(5.27)	103(6.45)	172(11.77)	<0.001	0.234
	Male	467(94.73)	1494(93.55)	1289(88.23)		0.234
Race	Black	98(19.88)	313(19.6)	244(16.7)	0.248	0.082
	Other/Unknown	29(5.88)	83(5.2)	81(5.54)		0.030
	White	366(74.24)	1201(75.2)	1136(77.75)		0.082
Age (mean, standard deviation)		69.02(11)	66.55(10.34)	60.63(15.34)	<0.001	0.629
Charlson comorbidity (mean, standard deviation)		3.26(2.75)	3.63(2.52)	1.16(1.74)	<0.001	1.140
Hypercholesterolemia		11(2.23)	55(3.44)	38(2.6)	0.236	0.073
Hyperlipidemia		303(61.46)	985(61.68)	622(42.57)	<0.001	0.390
Hypertriglyceridemia		65(13.18)	320(20.04)	183(12.53)	<0.001	0.204
Hypertension		387(78.5)	1382(86.54)	868(59.41)	<0.001	0.642
Smoking		88(17.85)	243(15.22)	216(14.78)	0.255	0.083
Ischemic Heart Disease		156(31.64)	516(32.31)	257(17.59)	<0.001	0.345
Non-ischemic Heart Disease		144(29.21)	375(23.48)	183(12.53)	<0.001	0.419
Metformin MPR*100 (mean, standard deviation) HbA1c (mean, standard deviation)		--(--)	0.73(0.28)	--(--)	--(--)	--(--)
		6.38(1.16)	7.64(1.63)	--(--)	<0.001	0.898
Index year (mean, standard deviation)		2017.3(1.64)	2017.39(1.59)	2017.37(1.61)	0.587	0.053
MPR = Medication procession ratio						

* *p*-value is from Chi-Square test or ANOVA comparing across cohorts.

**Table 2:** Overall mortality rates.

	Diabetics with No Metformin	Diabetics with Metformin	No Diabetes	*p*-Value	Standardized Difference
Overall Death Number of patients (%)	92(18.66)	198(12.4)	118(8.08)	<0.001	0.315

**Table 3:** Cox proportional hazards model for mortality.

	Mortality
Variables	HR (95% CI)
Diabetic status (reference = Non-Diabetic)	
Diabetic no metformin	1.046 (0.781–1.400)
Diabetic with metformin	0.780 (0.609–0.999)
Sex (reference = Female)	
Male	1.223 (0.735–2.034)
Age	1.048 (1.039–1.057)
Race (reference = White)	
Black	0.761 (0.576–1.007)
Other/Unknown	0.675 (0.378–1.204)
Charlson comorbidity	1.264 (1.221–1.308)
Pure Hypercholesterolemia	0.819 (0.459–1.461)
Hyperlipidemia	0.757 (0.612–0.936)
Hypertriglyceridemia	0.830 (0.629–1.094)
Hypertension	1.072 (0.803–1.433)
Smoking	1.365 (1.083–1.721)
Ischemic Heart Disease	0.974 (0.779–1.218)
Non-ischemic Heart Disease	1.397 (1.110–1.758)
Index year	0.896 (0.845–0.950)

## Data Availability

These analyses were performed using data that are available within the US Department of Veterans Affairs secure research environment, the VA Informatics and Computing Infrastructure (VINCI). All relevant data outputs are within the paper and its supplemental information.

## Supplementary Materials

The following supporting information can be downloaded at: https://www.mdpi.com/article/10.3390/pathogens11020270/s1, Table S1: Baseline Demographic and Comorbid Characteristics for Matched Cohort; Table S2: Mortality rate among matched cohorts; Table S3: Cox Proportional Hazards Model for Death in Matched Cohort; Table S4: Mortality rate at 30- and 60-days post influenza diagnosis among the original cohort; Table S5: Cox Proportional Hazards Model for 30-day and 60-day Mortality among the original cohort; Table S6: Diagnosis and LOINC Test Codes associated with confirmed laboratory influenza; Table S7: Baseline Demographic and Comorbid Characteristics for Cohort without Positive COVID test; Table S8: Mortality rate among matched cohort without Positive COVID test; Table S9: Cox Proportional Hazards Model for Death in Cohort without Positive COVID test.
