# Supplementary material for: Patients with Obesity and a History of Metformin Treatment Have Lower Influenza Mortality: A Retrospective Cohort Study
Source: Pathogens. 2022 Feb 19;11(2):270. doi: 10.3390/pathogens11020270 (PMC8876732; doi:10.3390/pathogens11020270)
Supplement: Supplementary file 1 [file pathogens-11-00270-s001.zip › pathogens-1512346-supplementary.pdf]

Table S1. Baseline Demographic and Comorbid Characteristics for Matched Cohort.

Variable		Diabetes				
		No Metformin N=456	Metformin exposed N=456	Non-Diabetes N=456	*p*-value	Standardized Difference
Sex	Female	25(5.48)	36(7.89)	33(7.24)	0.330	0.097
	Male	431(94.52)	420(92.11)	423(92.76)		0.097
Race	Black	92(20.18)	89(19.52)	86(18.86)	0.971	0.033
	Other/Unknown	28(6.14)	26(5.7)	30(6.58)		0.037
	White	336(73.68)	341(74.78)	340(74.56)		0.023
Age (mean, standard deviation)		67.94(10.48)	65.96(11.25)	66.86(13.13)	0.037	0.182
Charlson comorbidity (mean, standard deviation)		2.95(2.49)	2.74(2.15)	2.19(2.22)	<0.001	0.324
Pure Hypercholesterolemia		11(2.41)	14(3.07)	20(4.39)	0.235	0.109
Hyperlipidemia		271(59.43)	249(54.61)	260(57.02)	0.339	0.097
Hypertriglyceridemia		64(14.04)	71(15.57)	80(17.54)	0.345	0.096
Hypertension		357(78.29)	356(78.07)	377(82.68)	0.150	0.116
Smoking		75(16.45)	68(14.91)	62(13.6)	0.483	0.080
Ischemic Heart Disease		132(28.95)	133(29.17)	124(27.19)	0.769	0.044
Non-ischemic Heart Disease		109(23.9)	109(23.9)	109(23.9)	1.000	0.000
Metformin MPR*100 (mean, standard deviation)		--(--)	0.75(0.27)	--(--)	--(--)	--(--)
HbA1c (mean, standard deviation)		6.36(1.18)	7.63(1.6)	--(--)	<0.001	0.900
Index year (mean, standard deviation)		2017.29(1.62)	2017.35(1.73)	2017.12(1.72)	0.095	0.135

* *p*-value is from Chi-Square test or ANOVA comparing across cohorts.

Table S2. Mortality rate among matched cohorts.

	Diabetics with no metformin (N=456)	Diabetics with metformin (N=456)	No diabetes (N=456)	*p*-value	Standardized Difference
Number of patients (%)	74(16.23)	36(7.89)	57(12.5)	<0.001	0.258

Table S3. Cox Proportional Hazards Model for Death in Matched Cohort.

	Death
**Variables**	**HR (95% CI)**
Diabetic status (reference=Non-Diabetic)	
Diabetic no metformin	1.154 (0.805-1.654)
Diabetic with metformin	0.590 (0.384-0.905)
Sex (ref=Female)	
Male	1.188 (0.517-2.730)
Age	1.049 (1.034-1.064)
Race (reference=White)	
Black	1.070 (0.718-1.595)
Other/Unknown	0.959 (0.443-2.076)
Charlson comorbidity	1.265 (1.198-1.336)
Pure Hypercholesterolemia	0.633 (0.257-1.559)
Hyperlipidemia	0.789 (0.571-1.091)
Hypertriglyceridemia	0.967 (0.624-1.498)
Hypertension	0.925 (0.588-1.454)
Smoking	1.333 (0.916-1.942)
Ischemic Heart Disease	0.963 (0.676-1.372)
Non-ischemic Heart Disease	1.510 (1.069-2.133)
Index year	0.873 (0.799-0.954)

Table S4. Mortality rate at 30- and 60-days post influenza diagnosis among the original cohort.

	**Diabetics with no metformin (N=493)**	**Diabetics with metformin (N=1597)**	**No diabetes (N=1461)**	***p*-value**	**Standardized Difference**
30-day Mortality Number of patients (%)	7(1.42)	7(0.44)	5(0.34)	0.0139	0.115
60-day Mortality Number of patients (%)	10(2.03)	17(1.06)	7(0.48)	0.0079	0.139

Table S5. Cox Proportional Hazards Model for 30-day and 60-day Mortality among the original cohort.

Variables	30-day Death HR (95% CI)	60-day Death HR (95% CI)
Diabetic status (reference=Non-Diabetic)		
Diabetic no metformin	2.407 (0.705-8.217)	1.812 (0.640-5.130)
Diabetic with metformin	0.935 (0.274-3.191)	1.286 (0.502-3.292)
Sex (ref=Female)		
Male	na (na-na)*	na (na-na)*
Age	1.057 (1.014-1.102)	1.051 (1.019-1.084)
Race (reference=White)		
Black	0.280 (0.037-2.141)	0.527 (0.158-1.763)
Other/Unknown	1.035 (0.134-8.001)	0.679 (0.091-5.078)
Charlson comorbidity	1.129 (0.958-1.329)	1.244 (1.119-1.383)
Pure Hypercholesterolemia	na (na-na)*	na (na-na)*
Hyperlipidemia	0.649 (0.246-1.712)	0.625 (0.298-1.312)
Hypertriglyceridemia	0.979 (0.280-3.425)	0.622 (0.217-1.788)
Hypertension	0.863 (0.266-2.806)	0.479 (0.206-1.111)
Smoking	1.649 (0.568-4.785)	1.235 (0.504-2.824)
Ischemic Heart Disease	0.389 (0.128-1.179)	0.532 (0.248-1.143)
Non-ischemic Heart Disease	2.077 (0.712-6.058)	2.859 (1.262-6.480)
Index year	0.821 (0.643-1.049)	0.905 (0.740-1.106)

* Hazard ratio and confidence interval is not estimated due to sample size and few patients within categories in outcome of interest

Table S6. Diagnosis and LOINC Test Codes associated with confirmed laboratory influenza.

ICD9 Codes	078.2, 465.8, 465.9, 487.0, 487.1, 487.8, 488.01, 488.02, 488.09, 488.1, 488.11, 488.12, 488.19, 488.81, 488.82, 488.89, 780.60, 780.61, 780.64, 786.2, V04.81, V06.6
ICD10 Codes	A01.00, A37.00, A68.9, A78., A94., A98.8, G44.83, J06.9, J09.X1, J09.X2, J09.X3, J09.X9, J10.00, J10.01, J10.08, J10.1, J10.2, J10.81, J10.82, J10.83, J10.89, J11.00, J11.08, J11.1, J11.2, J11.81, J11.82, J11.83, J11.89, J12.2, J20.4, J45.991, R05., R06.6, R50.2, R50.81, R50.82, R50.9, R68.83
LOINC	34487-9, 38272-1, 40982-1, 48509-4, 49521-8, 49524-2, 49531-7, 53250-7, 53251-5, 55465-9, 60494-2, 62462-7, 76078-5, 76080-1, 77026-3, 77027-1, 77028-9, 82166-0, 82167-8, 82168-6, 82169-4, 82170-2

Table S7. Baseline Demographic and Comorbid Characteristics for Cohort without Positive COVID test.

Variable		Diabetes: No Metformin N=474	Diabetes: Metformin exposed N=1547	Non-Diabetes N=1424	*p*-value	Standardized Difference
Sex	Female	26(5.49)	100(6.46)	169(11.87)	<0.001	0.228
	Male	448(94.51)	1447(93.54)	1255(88.13)		0.228
Race	Black	92(19.41)	296(19.13)	236(16.57)	0.382	0.074
	Other/Unknown	26(5.49)	79(5.11)	81(5.69)		0.026
	White	356(75.11)	1172(75.76)	1107(77.74)		0.062
Age (mean, standard deviation)		69.08(11.05)	66.67(10.33)	60.63(15.35)	<0.001	0.632
Charlson comorbidity (mean, standard deviation)		3.26(2.76)	3.64(2.53)	1.16(1.74)	<0.001	1.141
Pure Hypercholesterolemia		9(1.9)	53(3.43)	37(2.6)	0.158	0.095
Hyperlipidemia		292(61.6)	952(61.54)	606(42.56)	<0.001	0.388
Hypertriglyceridemia		61(12.87)	312(20.17)	181(12.71)	<0.001	0.202
Hypertension		371(78.27)	1341(86.68)	848(59.55)	<0.001	0.643
Smoking		85(17.93)	234(15.13)	214(15.03)	0.279	0.078
Ischemic Heart Disease		150(31.65)	500(32.32)	252(17.7)	<0.001	0.343
Non-ischemic Heart Disease		138(29.11)	368(23.79)	178(12.5)	<0.001	0.418
Metformin MPR*100 (mean, standard deviation)		--(--)	0.73(0.28)	--(--)	--(--)	--(--)
HbA1c (mean, standard deviation)		6.37(1.17)	7.66(1.63)	--(--)	<0.001	0.906
Index year (mean, standard deviation)		2017.29(1.65)	2017.37(1.6)	2017.36(1.61)	0.605	0.052

Table S8. Mortality rate among matched cohort without Positive COVID test.

	Diabetics with no metformin (N=474)	Diabetics with metformin (N=1547)	No diabetes (N=1424)	p-value	Standardized Difference
Number of patients (%)	92(19.41)	197(12.73)	118(8.29)	<0.001	0.326

Table S9. Cox Proportional Hazards Model for Death in Cohort without Positive COVID test.

Variables	Death<br>HR (95% CI)
Diabetic status (reference=Non-Diabetic)	
Diabetic no metformin	1.053 (0.786-1.411)
Diabetic with metformin	0.778 (0.608-0.996)
Sex (ref=Female)	
Male	1.231 (0.740-2.048)
Age	1.048 (1.039-1.058)
Race (reference=White)	
Black	0.831 (0.628-1.100)
Other/Unknown	0.679 (0.380-1.211)
Charlson comorbidity	1.265 (1.222-1.309)
Pure Hypercholesterolemia	0.800 (0.448-1.428)
Hyperlipidemia	0.762 (0.617-0.942)
Hypertriglyceridemia	0.847 (0.642-1.117)
Hypertension	1.058 (0.792-1.413)
Smoking	1.403 (1.113-1.770)
Ischemic Heart Disease	0.986 (0.789-1.232)
Non-ishemic Heart Disease	1.404 (1.117-1.766)
Index year	0.898 (0.847-0.951)
